# From microscopy to molecular diagnostics: identifying the complexities of clinical diagnostics for vaginal infections

**DOI:** 10.1128/spectrum.00251-25

**Published:** 2025-08-20

**Authors:** Megan H. Amerson-Brown

**Affiliations:** 1 Department of Pathology, The University of Alabama at Birmingham 9968, Birmingham, Alabama, USA; London Health Sciences Centre, London, Ontario, Canada

**Keywords:** vaginal infections, candidiasis, bacterial vaginosis, trichomoniasis, clinical diagnostics

## Abstract

The use of molecular-based assays to diagnose vaginitis has become increasingly common. In a recent study, J. Elvy, K. Carter, J. Paterson, M. Smith, et al. (Microbiol Spectr 13: e0127424, 2025, https://doi.org/10.1128/spectrum.01274-24) compared the performance of the Hologic Panther Aptima BV and CV/TV assays for the diagnosis of vaginitis to that of the current standard-of-care diagnostic methods that include microscopy, culture, and PCR. The study suggests that the Hologic Panther Aptima BV and CV/TV assays are useful alternatives to conventional testing but would likely lead to higher treatment rates and additional testing costs. However, additional studies are needed to determine the clinical significance of these diagnostic assays, especially for BV and vulvovaginal candidiasis, wherein the presence or absence of specific organisms does not necessarily indicate clinical disease.

## INTRODUCTION

Diagnosing vaginitis is challenging since it can be caused by an imbalance of normal colonizing microbial organisms and not just simply the presence of a specific pathogen. Historically, vaginitis has been diagnosed using microscopy for vulvovaginal candidiasis (CV) and *Trichomonas vaginalis* (TV) and Amsel’s criteria or Nugent scoring for bacterial vaginosis (BV). The ability for rapid diagnosis by provider-performed wet-mount microscopy highlights the importance of treating these infections in a timely manner due to infection sequelae, loss to follow-up, and stigma surrounding vaginal infections. However, these traditional testing methods lack sensitivity and specificity, as some vaginal infections can mimic each other, and asymptomatic infections with BV and TV can lead to negative health outcomes (1, 2).

Despite Amsel’s criteria being the most common technique for diagnosing BV, the gold standard is Nugent scoring. Nugent scoring involves Gram staining, a vaginal swab to assess bacterial morphology and determine whether the content reflects normal vaginal flora or dysbiosis (2–4). Similarly, the simple presence or absence of yeast is also not clinically indicative of CV; yeast in vaginal samples should be assessed for morphology characteristics, quantity, and species identification (5, 6). *Trichomonas vaginalis* (TV) is an exception, as its presence is always considered pathogenic (3). However, the sensitivity of wet-mount microscopy for TV is low due to the need to visualize the living organism. Improved diagnostics, including molecular multiplex assays for vaginal infections, such as BV, CV, or TV, can reduce recurrence and improve health outcomes by providing results that lead to effective timely treatment and identification of co-infections when present. Improvements that are needed in vaginitis diagnostics include rapid turn-around times, improved targets for differentiating colonization and infection, and identifying antimicrobial resistance for select organisms (1, 7).

Novel molecular diagnostic assays with improved sensitivity and specificity, as evaluated in the recent study by Elvy et al., are likely to capture more infections than traditional methods, such as wet mounts and Amsel’s criteria (8). These assays do not address many of the reasons previously described, such as turn-around-time, as to why wet mounts and Amsel’s criteria were preferred in the clinical setting, and only if an infection cannot be determined are these rapid molecular diagnostics utilized. In addition, the molecular-based assays are more expensive than traditional microscopy techniques (1).

Elvy et al. recently studied the performance of two assays, Aptima BV and Aptima CV/TV assays, using the Hologic Panther System that utilizes transcription-mediated amplification to detect targets of interest. The study was performed in an accredited clinical reference laboratory that serves both community and hospitalized patients located in Te Waipounamu, the South Island of New Zealand, where the use of molecular-based assays for vaginitis is not currently the clinical standard (8).

The study involved 300 swabs submitted from symptomatic patients between 14 and 60 years old. Both assays were compared to current reference methods in use: Aptima BV assay was compared to consensus Gram stain and Nugent score, and Aptima CV/TV was compared to Gram stain and culture and the Aptima TV singleplex assay (8). Most of the patients included in the study were of European ethnicity; however, approximately 37% were either of native descent, undisclosed, or Asian/Pacific Islander ethnicity. Only 6% of the samples included in this study were from pregnant women (8). As such, both ethnicity-related genetics and hormonal changes as seen in pregnancy are known to impact the vaginal microbiome and BV incidence (9).

Overall, the assays evaluated in this study showed high sensitivity and specificity across all three causes of vaginitis: BV (97.5%; 96.3%), CV (100%; 83.5% with Gram stain, 96.3%; 91.2% with culture), and TV (100%; 100%) (8). The Aptima BV assay did not have a corresponding category to intermediate Nugent’s score results; therefore, these samples could not be assessed, although many of these patients were treated for BV based on the intermediate score results. The Aptima CV assay was highly sensitive but lacked specificity when compared to the Gram stain method, indicating that the comparison of performances is likely due to differences in diagnostic criteria and detection limitations (8). In addition, this study did not assess the accuracy of the assay to call the yeast the correct species. The target sequence for TV on both the reference and comparator assay was the same; therefore, the 100% correlation between the assays is not surprising (6, 8).

Interestingly, all commercially available assays for BV rely on a variety of different targets; however, all of them claim high sensitivity and specificity for diagnosing BV in symptomatic women (2). These differences indicate the limitations of vaginitis assays and our lack of understanding of the pathogenesis of BV infections (2). Similarly, while CV is better understood, limitations with assays, such as the inability to differentiate different *Candida species* or the limited number of species identified, can provide limited information as to the effectiveness of first-line treatment options, such as fluconazole (5).

Nucleic acid amplification tests (NAATs) can improve reproducibility and consistency of reporting for these individual infections, as a NAAT is less subjective than traditional diagnostic techniques for vaginitis. The findings from Elvy NAAT is less subjective than traditional diagnostic techniques for vaginitis. The findings from Elvy et al. indicated that molecular diagnostic assays have the potential to drastically improve the sensitivity of the clinical assays used to diagnose vaginal infection, including BV, CV, and TV; however, additional studies are needed surrounding BV and CV to indicate the exact parameters needed for clinical diagnostic molecular-based assays to consider a result “positive.” Similarly, the increased sensitivity for TV is clinically important. Novel point-of-care molecular diagnostics could be useful to provide quick turnaround times and the high sensitivity and specificity of molecular diagnostics when loss to follow-up is high (10).
